# Expression profiles of nestin and synemin in reactive astrocytes and Müller cells following retinal injury: a comparison with glial fibrillar acidic protein and vimentin

**Published:** 2010-11-27

**Authors:** Gabriel Luna, Geoffrey P. Lewis, Christopher D. Banna, Omar Skalli, Steven K. Fisher

**Affiliations:** 1 Neuroscience Research Institute, University of California Santa Barbara, Santa Barbara, CA; 2 Department of Molecular, Cellular, and Developmental Biology, University of California Santa Barbara, Santa Barbara, CA; 3 Department of Biological Sciences, University of Memphis, Memphis TN

## Abstract

**Purpose:**

To examine the expression patterns of the intermediate filament (IF) proteins nestin and synemin following retinal injury.

**Methods:**

Wide-scale retinal injuries were created by experimental retinal detachment of 1, 3, 7, or 30 days’ duration. Injuries were induced in the right eyes of Long Evans rats, while the left eyes served as internal controls. Vibratome sections of control and injured retinas were labeled with fluorescent probes using a combination of anti–glial fibrillary acidic protein, -vimentin, -nestin, -synemin, -bromodeoxyuridine, and the lectin probe, isolectin B4. Additionally, antibody specificity, as well as protein and mRNA levels of nestin and synemin were determined and quantified using standard western blotting and real time polymerase chain reaction (RT–PCR) techniques.

**Results:**

Immunocytochemistry showed increased Müller cell labeling at 1, 3, and 7 days post injury for all four IFs, although the relative levels of nestin expression varied dramatically between individual Müller cells. Nestin was consistently observed in the foremost processes of those Müller cells that grew into the subretinal space, forming glial scars. Elevated levels of nestin expression were also observed in bromodeoxyuridine-labeled Müller cells following retinal insult. Quantitative polymerase chain reaction (qPCR) showed a twofold increase in nestin mRNA 1 day after injury, a level maintained at 3 and 7 days. Western blotting using anti-nestin showed a single band at 220 kDa and the intensity of this band increased following injury. Anti-synemin labeling of control retinas revealed faint labeling of astrocytes; this increased after injury, demonstrating an association with blood vessels. Additionally, there was an upregulation of synemin in Müller cells. qPCR and western blotting with anti-synemin showed a continuous increase in both gene and protein expression over time.

**Conclusions:**

Retinal injury induces an upregulation of a complement of four intermediate filament proteins, including synemin and nestin, in Müller cells. The latter provides suggestive support for the concept that these cells may revert to a more developmentally immature state, since these two IF proteins are developmentally regulated and expressed, and thus may serve as cell cycle reentry markers. Nestin and its differential expression patterns with glial fibrillary acidic protein and vimentin networks, as well as its association with proliferating Müller cells and those extending into the subretinal space, suggest a significant role of this protein in glial scar formation and perhaps gliogenesis. Synemin immunopositive astrocytes demonstrate a close relationship to the retinal vasculature, and illustrate a remarkable ability to reorganize their morphology in response to injury. Further examination of the changes in the cytoskeletal signatures of both of these glial cell types may lead to a more comprehensive understanding of mechanisms underway following retinal and other central nervous system injuries.

## Introduction

Intermediate filaments (IF) are abundant and dynamic cytoskeletal components in eukaryotic cells [1]. Approximately 70 genes encode eukaryotic IF proteins, ranking this protein family among the largest in humans [2,3]. Structurally, all members of this family contain a non-α-helical N-terminal head and a C-terminal tail domain flanking a central rod domain [4,5]. A common feature of all IF proteins is their ability to form homo- or heteropolymers, a physical property that results in a large number of IF protein partnerships [6]. In general, IF proteins are expressed in a cell-, tissue-, and developmentally specific manner, thereby creating a “cytoskeletal fingerprint” that can provide insight into cellular dynamics, such as differentiation, reactivity, or the mitotic state [7].

Synemin and nestin are developmentally regulated IF proteins that are expressed in undifferentiated astrocytes, as well as in a subset of cortical glia [8]. Synemin, also known as desmuslin, has two unique features: the occurrence of three splice variants (H, M, and L), and the capacity to interact with actin-associated proteins [9–11]. The major developmental cytoskeletal protein nestin is also expressed during neural development, but is ultimately downregulated and replaced by glial fibrillary acidic protein (GFAP) in astrocytes and vimentin in Müller cells, the specialized radial glial cells of the retina.

In the vertebrate retina, Müller’s glia account for approximately 90% of the glial population [12] and exhibit a complex structural architecture spanning the entire retina from the nerve fiber layer (NFL) to the base of the photoreceptor inner segments. Retinal astrocytes are only found in the NFL and account for 10% of the glial population. Together, astrocytes and Müller cells perform a multitude of developmental and physiologic functions, including supplying metabolic support by means of glutamine and taurine in the normal retina [13], maintaining K^+^, H^+^, and water balance, protecting against oxidative stress, recycling cone photopigments, releasing neuro- and vasoactive substances, and serving as scaffolds for neurovascular guidance [14,15]. Under normal conditions, rodent retinal Müller cells predominately express vimentin. After injury, however, they undergo a rapid change in the composition of their cytoskeleton with the upregulation of both vimentin and GFAP, a response indicative of a state of reactivity [16,17].

Collectively, Müller cells and astrocytes comprise the intricate glial network of the retina [18], and both have been shown to have similarities to glia in other parts of the central nervous system (CNS) [19–21]. The cellular blueprint of both retinal astrocytes and Müller cells creates a complex ordered grid, providing essentially complete glial coverage of the entire neural retina. However, unlike Müller cells, which span several retinal layers, astrocytes reside only in the innermost layer of the retina, the NFL. In rodents, astrocytes arrive at their final destination during development by migrating through the optic nerve during the first postnatal week. In the rat retina, astrocytes demonstrate fibrous or stellate morphology, possessing a highly planar appearance in contrast to the classical protoplasmic morphology of cortical gray matter astrocytes [22]. Astrocytes also serve a vital role in species that contain a vascular retina by secreting trophic factors such as vascular endothelial growth factor, which permits and stimulates the formation of the retinal vascular network [18].

After injury, Müller cells participate in a phenomenon known as reactive gliosis, where the term “reactive” encompasses a wide range of molecular, biochemical, and morphologic events. Anatomically, this process leads to an increase in the size of Müller cells, as well as their growth out of the neural retina, producing a scar that creates a physical barrier between the retinal pigmented epithelium (RPE) and photoreceptors, ultimately preventing the regeneration of damaged photosensitive outer segments following retinal reattachment [23]. Reactive gliosis is easily demonstrated by immunocytochemistry due to the increased expression of GFAP and vimentin in Müller cells [17,24]. Other Müller cell responses to injury-induced retinal degeneration include the induction of proliferation [25–27] and the downregulation of specific enzymes and molecules vital to retinal homeostasis such as glutamine synthetase, carbonic anhydrase C, and cellular retinaldehyde binding protein [28].

The dynamic relationship between IFs and retinal glia may underscore a key mechanism in the rapid modification of Müller cell structure in response to retinal injury or other changes in the retinal milieu [29]. Indeed, in GFAP^-^/^-^ vimentin^-^/^-^ mice, Müller cells are unable to hypertrophy or extend processes outside the neural retina following injury [30]. In fact, studies of these mice suggest that the absence of these two IF proteins may be neuroprotective following retinal injury [31]. The goal of our study was to determine whether the two developmentally regulated IF proteins nestin and synemin also show increased expression in the adult after retinal injury, and if so, to determine their affiliation and distribution within the retina relative to that of vimentin and GFAP.

## Methods

### Surgery

This study was conducted in accordance with the NIH Animal Care and Use Committee protocols, the ARVO Statement for the Use of Animals in Ophthalmic and Vision Research, as well as the guidelines of the Animal Resource Center of the University of California Santa Barbara (Santa Barbara, CA). Animals were housed in a 12 h light-dark cycle and were provided food and water ad libitum. Using an operating microscope, retinal injuries were created in the right eyes of Long Evans rats (*Rattus norvegicus*) as described in detail elsewhere [28,30], while left eyes served as unoperated controls in all procedures. In short, a small incision was made to create a pilot hole through the sclera a few millimeters below the limbus. A pulled glass micropipette (approximately 50 μm in diameter), under the control of a micromanipulator, was inserted transvitreally between the neural retina and the underlying RPE. A glass coverslip, placed on the cornea, was used to enhance the visualization of the retina during this procedure. Approximately 5 µl of solution containing Healon (0.25% sodium hyaluronate; Pharmacia & Upjohn, Uppsala, Sweden) diluted in balanced salt solution (BSS; Alcon, Ft. Worth, TX) was infused between the neural retina and the RPE, detaching approximately 75% of the retina. The Healon was necessary to prevent the retina from spontaneously reattaching. No damage to the lens was observed at any time during the surgery or during dissection of the eyes. Retinal tissue from three animals per experimental group was harvested at 1, 3, 7, or 30 days following injury and processed for immunocytochemistry, PCR, and western blotting. Three additional rats were given an intravitreal injection of bromodeoxyuridine (BrdU; 10 µg in 10 µl of BSS; 4 h before sacrifice at the 3 day time point; Sigma, St. Louis, MO).

### Tissue preparation and fixation

Following euthanasia, globes were carefully enucleated and immersion fixed overnight in 4% paraformaldehyde in sodium cacodylate buffer (0.1 M; pH 7.4) at 4 °C. The following day, the cornea and lens were dissected to form an eye cup in preparation for agarose embedding and radial vibratome sectioning. Due to the sensitivity of the synemin epitope to fixation, lightly fixed cryosectioned tissue was needed. Here, eyes were excised as described above; however, after 90 min of immersion fixation, the cornea and lens were removed and the eye cup reimmersed in fixative for 30 min.

### Antibodies and immunocytochemistry (vibratome sections)

Immunocytochemical experiments were performed as described previously [32]. Following fixation, rodent globes were rinsed in PBS (pH 7.4) 3× for 15 min and once for 60 min, embedded in low-melt agarose (5%; Sigma) at 45 °C and then sectioned at 100 μm using a vibratome (Leica, Lumberton, NJ). For injured retinas, care was taken only to sample those regions observed to be elevated from the RPE at the time of enucleation. To prevent nonspecific binding of antibodies, retinal sections were blocked overnight with normal donkey serum 1:20 in PBS containing 0.5% BSA, 0.1% Triton X-100, and 0.1% Azide (PBTA) at 4 °C and placed on a rotator for continuous agitation. Primary antibodies (Table 1), diluted in PBTA, were added to the sections for another overnight incubation at 4 °C. The next day retinal sections were rinsed in PBTA, 3× for 15 min and once for 60 min, at which time the corresponding secondary antibodies (Table 1) were added for the last overnight incubation at 4 °C. Finally, the sections were rinsed in PBTA as described above, mounted in 5% n-propyl gallate in glycerol on glass slides, covered with a coverslip, and sealed with nail polish. Sections labeled with BrdU were preincubated in 2N HCl for 1 h at 4 °C as an antigen retrieval step then subsequently labeled using the above-mentioned protocol.

**Table 1:** Antibodies used for this study along with their source and concentrations.

**Antibody**	**Species**	**Form**	**Dilution**	**Manufacturer**
anti-vimentin	chicken polyclonal	purified immunoglobulin	1:2000	Millipore, Temecula CA
anti-GFAP	rabbit polyclonal	purified immunoglobulin	1:400	DAKO, Carpenteria, CA
anti-nestin	mouse monoclonal	purified immunoglobulin	1:50	Millipore, Temecula CA
anti-synemin	rabbit polyclonal	affinity purified immunoglobulin	1:20	[38]
anti-BrdU	rat monoclonal	purified immunoglobulin	1:250	Accurate cheminal, Westbury, NY
isolectin B4	lectin	biotinylated	1:50	Vector Labs, Burlingame, CA
**Secondary antibodies**				
Donkey anti-rabbit	Cy5	purified antisera	1:200	Jackson Immuno Research, West Grove, PA
Donkey anti-mouse	Cy3	purified antisera	1:200	Jackson Immuno Research, West Grove, PA
Donkey anti-chicken	Cy2	purified antisera	1:200	Jackson Immuno Research, West Grove, PA
Donkey anti-rat	Cy2	purified antisera	1:200	Jackson Immuno Research, West Grove, PA
Streptavidin	Cy5	purified antisera	1:200	Jackson Immuno Research, West Grove, PA

### Immunocytochemistry: cryosections

To produce cryosections, eye cups were rinsed 3× for 15 min in PBS and then cryoprotected overnight in 20% sucrose and 0.1 M phosphate buffer in Optimal Cutting Temperature medium (Electron Microscopy Sciences, Washington, PA), pH 7.4 at 4 °C. The next day, eyecups were embedded in 100% Optimal Cutting Temperature and quickly immersed in liquid nitrogen; 20 µm thick sections were cut using a cryostat (Leica). After collecting sections on a charged glass slide, sections were blocked overnight in a humid chamber in normal donkey serum diluted 1:20 in PBTA at room temperature. The next day, without rinsing, primary antibodies (Table 1) diluted in PBTA were added to the sections and incubated overnight. Sections were then rinsed in PBTA 3× for 15 min and once for 1 h, at which time they underwent a final incubation in a solution of PBTA containing the appropriate combination of secondary antibodies (Table 1) for 2 h at room temperature. After rinsing in PBTA, sections were mounted and sealed as described above.

### Image acquisition

The specimens were viewed and images collected on an Olympus FluoView 1000 laser scanning confocal microscope (Center Valley, PA) using an UPlanFLN 40× oil immersion lens, N.A. 1.30. Optical sections were collected at 1 µm intervals and subsequently used to create maximum intensity projections using Olympus FluoView viewer version 1.7a. Confocal images were collected at an original pixel resolution of 1024×1024. Brightness and contrast levels were equally adjusted on all images using the bio-image analysis software Mayachitra Imago version 1.0 (Santa Barbara, CA) or Adobe Photoshop CS3 software (San Jose, CA).

### Quantitative real time polymerase chain reaction

Real-time (RT)–PCR was preformed as described in Radeke et al. [33]. To summarize, total RNA was isolated from rat tissue using Qiagen RNeasy mini-preps (Qiagen, Valencia, CA). To prevent DNA contamination, a DNA digestion step was included using RNase-free DNase (Ambion, Austin, TX). Subsequently, RNA samples were repurified, and the quality was examined by microchannel electrophoresis using an Agilent Bioanalyzer (Agilent Technologies Inc., Palo Alto, CA). Real-time quantitative PCR was performed using the SYBR Green method [34]. In short, primer pairs were designed to generate a 100–200 base pair product from a single site located no more than 600 base pairs away from the polyadenylated tail of the mRNA (mRNA; Beacon Design 4.0; Premier Biosoft International, Palo Alto, CA).

Using the iScript cDNA synthesis kit (Bio-Rad Laboratories, Richmond, CA), 250 ng of RNA at each time point were used to produce cDNA. Triplicate 20 µl PCR reactions were set up using the following reaction settings: 50 mM KCl, 10 mM Tris-HCl (pH 9.0 at 25 °C), 2.5 mM MgCl_2_, 0.1% Triton X-100, 0.2 mM deoxynucleotide triphosphate (dNTPs), 0.20 units of Platinum Taq DNA polymerase, 1× SYBR Green (Invitrogen Corp., Carlsbad, CA), and 500 nM of sense and antisense gene-specific primers (Operon, Huntsville, AL). Amplification of cDNA was performed on a Bio-Rad MyIQ Single Color Real-Time PCR detection system. The following temperature settings were used to carry out RT–PCR: 3 min at 95 °C, then 45 cycles of 15 s at 93 °C, 10 s at 56 °C, 90 s at 72 °C, 20 s at 78 °C, and 20 s at 82 °C. Real-time fluorescence readings were measured at 72 °C, 78 °C, and 82 °C. To decrease nonspecific signals due to mispriming or primer-dimer formation, real-time fluorescence measurements close to the melting temperature of the amplicons were used for quantitative analysis. The specificity of each reaction was assessed by melting temperatures and agarose gel electrophoresis of PCR products. To control for differences in amounts of starting material, the data were normalized to the geometric mean of a set of “housekeeping” genes with expression levels that have been empirically shown not to change as a function of location, age, or disease [35]. In this study, four housekeeping genes were used to quantify levels of mRNA for genes of interest (Table 2).

**Table 2:** Genes and their primer sequences used for quantitative PCR.

**Gene name**	**Gene symbol**	**Accession number**	**Primer sequence (5′-3′)**
glial fibrillary acidic protein	*Gfap*	NM_017009	F: GAGTCCACAACCATCCTTCTGAG
			R: ACACCAGGCTGCTTGAACAC
vimentin	*Vim*	NM_031140	F: CTGCTGGAAGGGGAGGAGAG
			R: GGTCATCGTGGTGCTGAGAAG
nestin	*Nes*	NM_012987	F: GTCCTGGTTCCTGAACTTGTC
			R: GCTTCTTTCTCTACCAGTTCCC
synemin	*Synm*	NM_001134858	F: AAGAAAGCAGTT TGAACAGAAAG
			R: TGGTGATTCCAACTTAGAGATGAC
glyceraldehyde phosphate dehydrogenase	*Gapdh*	NM_017008	F: AAGTTCAACGGCAGTCAAG
			R: ACTCAGCACCAGCTCACC
glucose phosphate isomerase	*Gpi*	NM_207592	F: AGACCATCACCAACGCAGAG
			R: CCACCTACCCAATCCCAGAAC
TATA box binding protein	*Tbp*	NM_001004198	F: CAGTCCAATGATGCCTTACGG
			R: TGTTGCTGCTGCTGTTGC
ubiquitin C	*Ubc*	NM_017314	F: GACAGGCAAGACCATCACTC
			R: CCAAGAACAAGCACAAGAAGG

### Western blots

Immunoblots were performed as described elsewhere in detail [36]. In brief, after the careful dissection of retinas from the eye cups, retinal tissue was flash frozen in liquid nitrogen until all time point samples were collected. Tissue was homogenized at 4 °C in cold PBS containing a protease inhibitor cocktail (Roche, Palo Alto, CA). Tissue lysates were then centrifuged at 11,000× g at 4 °C for 20 min, after which soluble supernatant material was collected and protein concentrations were determined using a DC protein assay kit (Bio-Rad Laboratories, Richmond, CA). Equal amounts of soluble protein were loaded into 7% polyacrylamide resolving gels and subsequently run at 100V for 90 min. Proteins were then electrophoretically transferred to a nitrocellulose membrane in sodium bicarbonate (10 mM NaHCO_3_, 3 mM Na_2_CO_3_, 10% methanol) buffer, blocked in PBS containing 5% dry nonfat milk and 0.1% Tween-20 (blocking buffer) overnight at 4 °C. The following day, blots were immunostained with primary antibodies, and diluted in blocking buffer containing 0.1% Tween-20 overnight at 4 °C. Blots were then rinsed 3× for 15 min in PBS and once for 1 h in PBS containing 0.1% Tween-20 (rinse buffer); then they were incubated in donkey anti-mouse, anti-chicken, or anti-rabbit Alexa Fluor 680 nm or 800 nm conjugated secondary antibodies (Molecular Probes, Eugene, OR) in blocking buffer containing 0.1% Tween-20 for 1 h at room temperature. After rinsing, blots were imaged using an Odyssey scanner (Li-Cor, Lincoln, NE).

## Results

### Müller cell responses: nestin, GFAP, and vimentin expression

**Figure 1:**
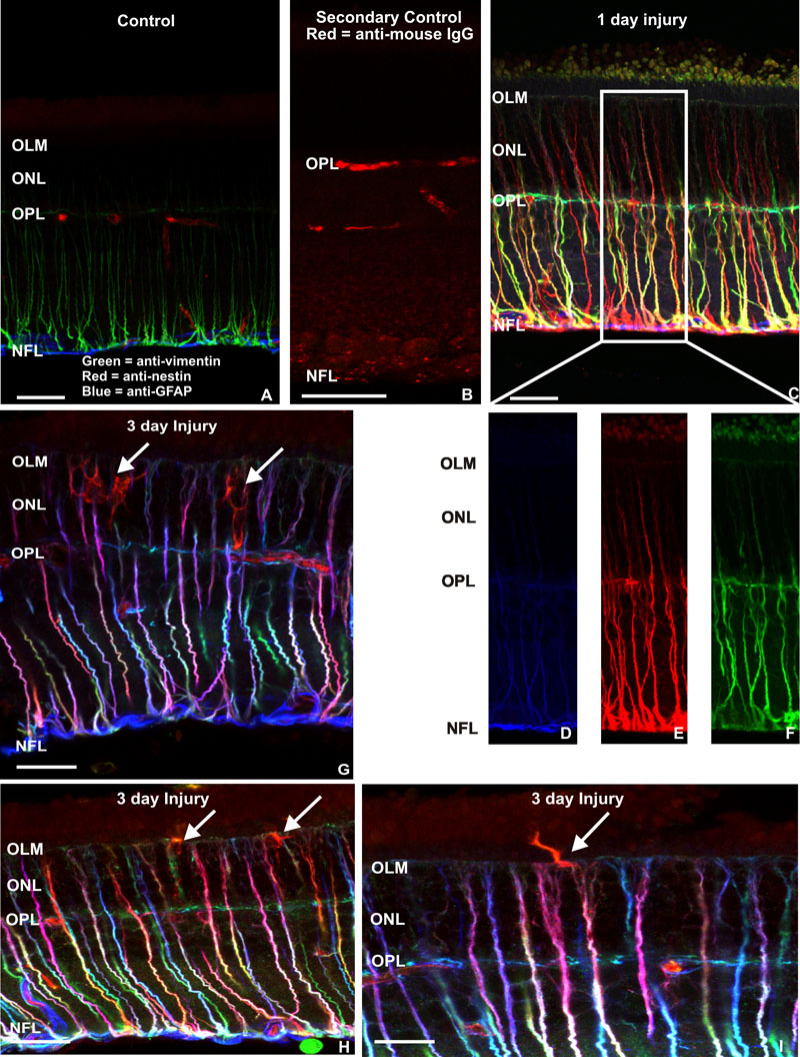
Laser scanning confocal images of control and injured retinas labeled with antibodies to nestin, glial fibrillary acidic protein, and vimentin. **A**, **B**: In the noninjured retina, glial fibrillary acidic protein (GFAP, blue) immunoreactivity is restricted to the very thin layer of astrocytes in the nerve fiber layer (NFL). Apparent nestin labeling (red) in vascular structures is due to non-specific binding of the mouse secondary IgG to rat blood vessels as demonstrated by the secondary control data in **B**. Anti-vimentin (green) labels all of the Müller cells from the NFL into the outer nuclear layer (ONL). **C**: Following 1 day of injury these three intermediate filament proteins are greatly upregulated with the labeling appearing as streaks extending across the retina. Note the heterogeneity of labeling patterns among Müller cells. **D**-**F**: Data shown in **C** is divided into its three RGB channels to demonstrate the distinctive increases in anti-nestin (**E**) and anti-vimentin (**F**) labeling relative to anti-GFAP (**D**). **G**, **H**, **I**: Three days following injury, the Müller cell labeling pattern appears distinctly different from those at 1 day as GFAP (blue) labeling increases, although the heterogeneity of intermediate filament protein labeling remains. Strongly nestin-positive cells resembling microglial cells in the ONL occur at this time point (**G**, arrows), while Müller cells expressing predominately nestin begin to show the formation of glial scars in the subretinal space (**I**, arrow). Scale bars represent 20 μm. OPL represents outer plexiform layer; OLM represents outer limiting membrane.

In control rat retinas, no specific nestin staining was detected in any cell type, although blood vessels in the outer plexiform layer (OPL) and in the NFL were nonspecifically immunolabeled (Figure 1A, red). Control staining with secondary antibodies alone showed that blood vessel labeling in control tissue was due to the secondary anti-rodent antibody recognizing IgGs in the blood vessels of the rat retina (Figure 1B). Anti-GFAP labeled only astrocytes in control retinas (Figure 1A; blue), while anti-vimentin labeled Müller cells from their endfeet in the NFL to the level of adherens junctions at the base of the photoreceptor inner segments (outer limiting membrane, OLM; Figure 1A; green). One day after injury, Müller cells displayed a dramatic increase in immunofluorescence, with nestin, GFAP, and vimentin labeling now extending from their endfeet in the NFL to the OLM (Figure 1C-F). The fact that most Müller cells produced yellow and orange signals 1 day after retinal injury indicates the robust coexpression of nestin (red) and vimentin (green) relative to GFAP (blue) at that time. Three days after injury, however, a change in the combinatorial expression profiles of IF proteins is evident in Müller cells, reflecting the vigorous upregulation of GFAP. At this time point, more Müller cells express increased levels of GFAP (blue) and nestin (red), producing signals that appear more pink or purple. In addition, nestin is expressed differentially among individual Müller cells relative to vimentin and GFAP, as shown by the resulting myriad of color combinations (Figure 1C,G,H,I). Interestingly, 3 days following injury, anti-nestin labeled rare complex processes in the ONL with the appearance of microglial cells (Figure 1G,H arrows). At this time, the initial stages of Müller cell growth into the subretinal space were observed as small tapered angular processes tipped with fine protuberances that were nestin positive (Figure 1I, arrow).

**Figure 2:**
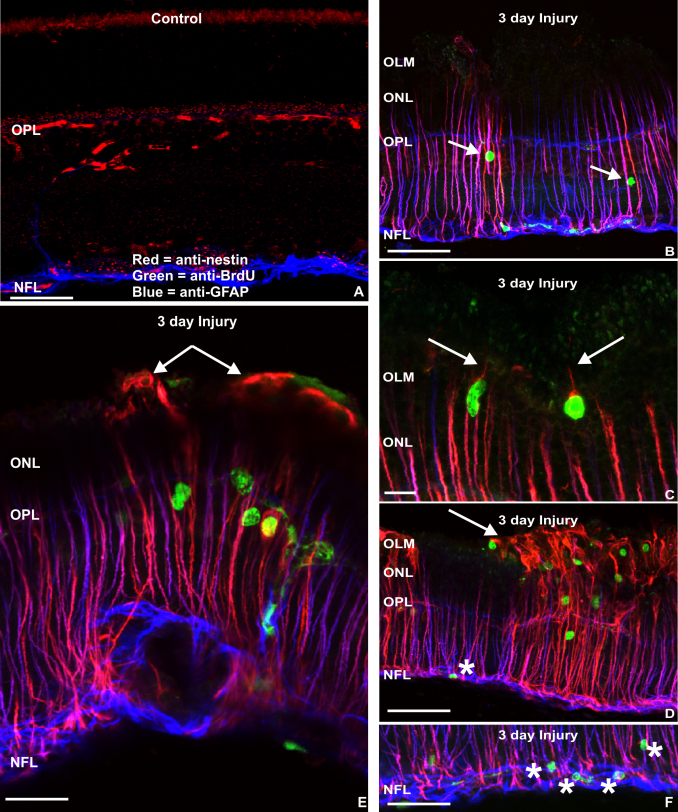
Laser scanning confocal images from control and injured retinas labeled with antibodies to nestin, bromodeoxyuridine and glial fibrillary acidic protein. **A**: In control retinas, apparent anti-nestin labeling (red) due to nonspecific binding of the anti-mouse secondary antibody (see Figure 1B) was observed outlining blood vessels in the outer plexiform layer, while anti-glial fibrillary acidic protein (GFAP, blue) strongly labeled astrocytes in the nerve fiber layer (NFL). **B**-**F**: After 3 days of a sustained retinal injury, anti-nestin labeling is more intense and more extensive in all Müller cells, but the degree of intensity varies across the retina as shown in figures **B** and **D**. Müller cell nuclei that incorporate bromodeoxyuridine (BrdU, green) are associated with areas of strong anti-nestin labeling in both figures **B** and **D**. Anti-nestin labeling of Müller cells is strongly associated with the presence of Müller cell outgrowth, whether at the earliest (**C**, arrows), or at more advanced stages of glial scar formation (**E**, **D**). In some cases, anti-BrdU labeled nuclei were observed in anti-GFAP labeled cells in the NFL indicating that astrocytic proliferation is also a component of the injury response (**D**, **F**, asterisks). In this study anti-nestin labeling was not observed in astrocytes. Scale bars represent 20 μm. OLM represents outer limiting membrane; ONL represents outer nuclear layer; OPL represents outer plexiform layer.

To determine whether there was an association between dividing Müller cells and IF protein expression, sections were labeled with anti-BrdU at day 3, a time point previously shown to be the peak of the proliferative response following retinal detachment [25,26]. In control, noninjured retinas, no BrdU labeling was observed; anti-GFAP labeled only astrocytes in the NFL, while the only apparent anti-nestin labeling represents binding of the secondary antibody to blood vessels (Figure 2A). Three days after injury, and 4 h after an intraocular injection of BrdU, BrdU-labeled Müller cell nuclei were observed in the inner nuclear layer (Figure 2B). These BrdU-labeled cells were consistently present in regions where Müller cells extended short processes beyond the OLM (Figure 2C; arrows), and in regions with significant growth of Müller cell processes into the subretinal space (Figure 2E,D; arrows). At this time, anti-BrdU labeled nuclei were also occasionally observed in astrocytes in the NFL (Figure 2D,F; asterisks). These presumed astrocytes, however, were never labeled with anti-nestin.

### Müller cells and astrocytes: synemin expression

**Figure 3:**
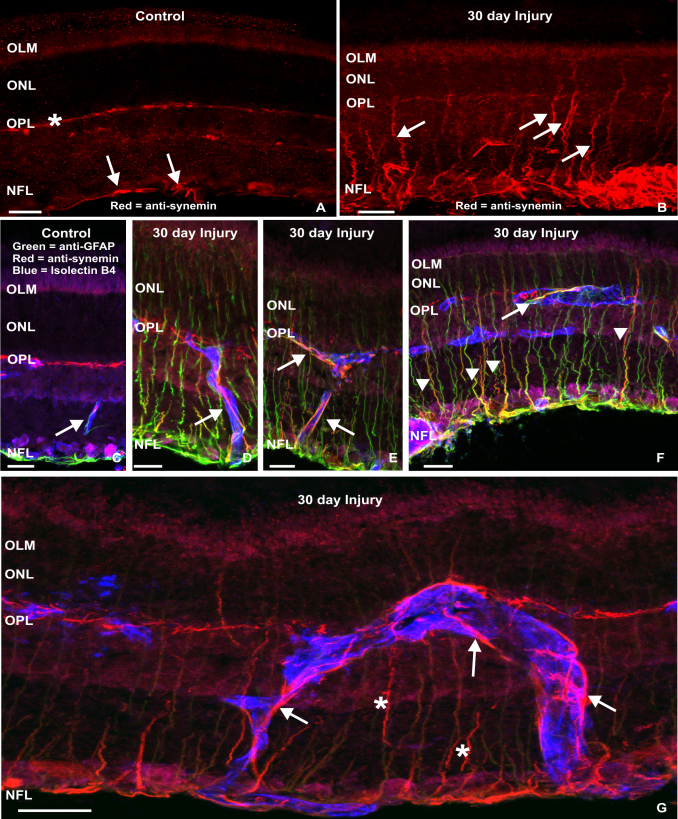
Laser scanning confocal images illustrating immunoreactivity for synemin, glial fibrillary acidic protein, and the isolectin B4 labeling in cryosections of control retina and following 30 days of injury. **A**: In control tissue, sparse synemin immunoreactivity (red) is present in astrocytes of the nerve fiber layer (NFL; arrows), as well as in cells along the inner border of outer plexiform layer (OPL, asterisk), perhaps representing the labeling of horizontal cells. **B**-**G**: After injury, anti-synemin labeling appears as vertical streaks across the retina representing the processes of Müller cells (**B**, arrows) as well as more intense labeling of astrocytes extending processes laterally in the NFL (**B**). The triple-labeled control retina shows the presence of anti-glial fibrillary acidic protein (GFAP, green) labeled astrocytes in the NFL, and an isolectin B4-positive blood vessel (blue, arrow) coursing across the inner retina (**C**). Following long-term injury, many Müller cells express synemin from the inner retina to the outer limiting membrane (OLM, **D**-**G**), although this population shows little overlap with radial processes expressing GFAP at this time. Anti-synemin labeling also increased in astrocytes after injury, now extending into the outer retina but still associated with retinal blood vessels (**D**-**G**, arrows). Scale bars represent 20 μm. ONL represents outer nuclear layer; OPL represents outer plexiform layer.

Control rat retinas displayed low levels of synemin labeling in presumptive horizontal cells in the OPL (Figure 3A,C, asterisk) and astrocytes in the NFL (Figure 3A, arrows). After 30 days of a sustained retinal injury, synemin immunoreactivity increased in Müller cells (Figure 3B, arrows) and in the NFL. At this time point, synemin antibodies labeled several Müller cells in a pattern similar to that for the other three IF proteins, with labeling through the length of the cell (Figure 3B-G), although this occurred with less frequency among the Müller cells in comparison to GFAP. Synemin labeling in the NFL of injured retinas that did not resemble that of Müller cells was consistently associated with the retinal vasculature, as identified by isolectin B4 labeling (Figure 3D-G, blue, arrows). These synemin-labeled processes often colocalized with anti-GFAP, and could be traced back to the NFL. In addition, these processes tended to contact and wrap blood vessels in a pattern more consistent with that of astrocytes than the “endfoot” type of contact made by the lateral processes of Müller cells. Interestingly, a small subpopulation of Müller cells apparently express synemin with little to no GFAP expression detected (Figure 3F, arrowheads), once again demonstrating the phenotypic diversity of Müller cells with respect to changes in the composition of their cytoskeleton as they respond to injury.

### Real time polymerase chain reaction: nestin and synemin

**Figure 4:**
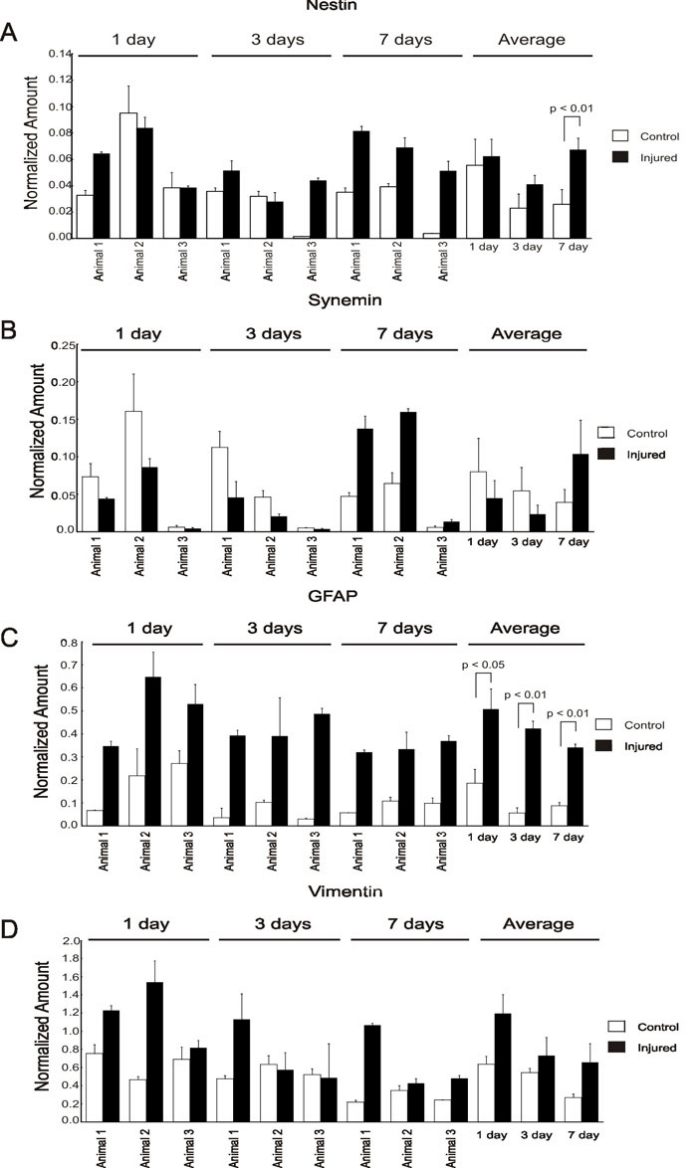
Real-time quantitative PCR gene expression analysis for control (left eyes, white bars) and injured retinas (right eyes, black bars). For each graph, the levels of gene expression for nestin (**A**), synemin (**B**), glial fibrillary acidic protein (GFAP; **C**), and vimentin (**D**) were normalized relative to the geometric mean of the four housekeeping genes: glyceraldehyde phosphate dehydrogenase, glucose phosphate isomerase, TATA box binding protein, and ubiquitin C. In general, mRNA levels were elevated at all injury time points. The p value shown for the average data was computed using a paired two-tailed Student *t* test (n=3). In this study, values of 0.01 or higher were considered statistically significant. Error bars denote standard deviations (SD).

RT–PCR demonstrated that the gene expression levels for nestin, GFAP, and vimentin all increased within a day after injury and stayed elevated until the 7 day time point. Nestin mRNA levels demonstrated an average twofold increase 7 days after injury (Figure 4A). Although immunocytochemical data showed a clear increase in nestin expression, PCR data showed an increase in mRNA levels at 1, 3, and 7 days with only the last reaching statistical significance. In comparison, RT–PCR data demonstrated that synemin mRNA levels were not statistically significantly different from those of the control (Figure 4B) at all time points, although they increased twofold at 7 days postinjury. This is in contrast to GFAP, which shows a rapid increase at 1 day that is sustained at 3 and 7 days after injury (Figure 4C). The pattern of vimentin mRNA in injured retinas compared to controls (Figure 4D) showed similar trends to that of GFAP, although the difference did not reach statistical significance, perhaps because of the relatively high levels of vimentin found in uninjured retinas.

### Western blots: nestin and synemin

**Figure 5:**
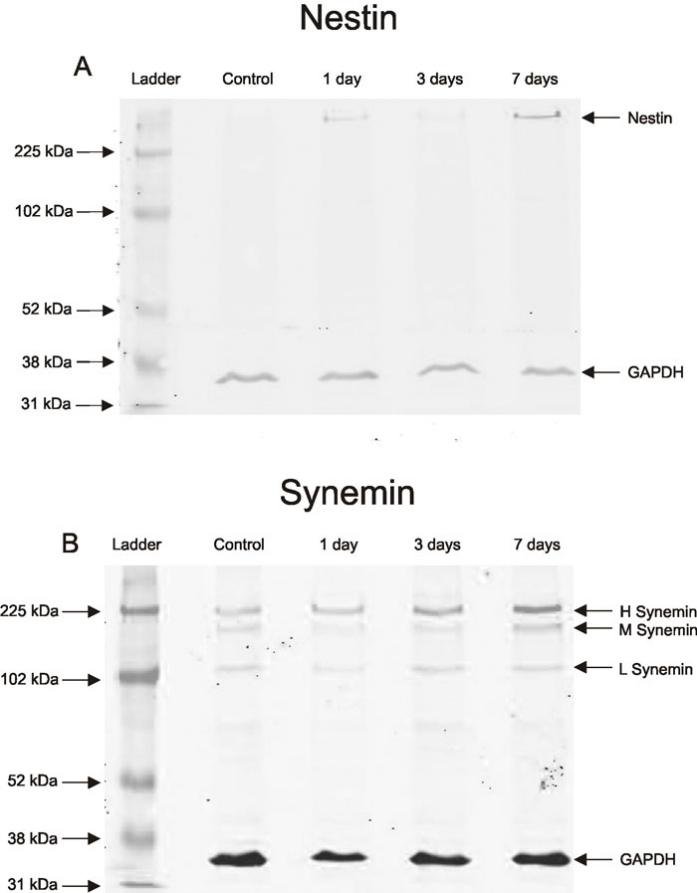
Immunoblot data for nestin and synemin. **A:** Very faint bands of nestin were visible at 1 day and this increased at 7 days; no band was observed in control retinas (250 kDa). Only one band at the appropriate molecular weight for nestin was observed, confirming antibody specificity. Lightly labeled synemin bands appeared in control tissue with a steady increase between 1 and 3 days of injury and a strong increase by 7 days (arrows). **B**: Two primary bands (arrows) were detected for synemin, corresponding to the presence and specificity of the antibody for both the H and M isoforms (225 kDa, 175 kDa). The internal control glyceraldehyde phosphate dehydrogenase (35 kDa) was used to ensure equal protein loading.

Western blotting detected no nestin in the control retinas (Figure 5A), but faint bands were observed at the 1 and 7 day time points. The single band at approximately 250 kDa confirmed antibody specificity and demonstrated an increase from control retinas. Western blotting also showed an increase in both the H and M isoforms of synemin protein after 7 days of injury (Figure 5B), with a more dramatic increase in the H isoform.

## Discussion

In this study, we demonstrate that expression levels of the IF protein synemin, in addition to GFAP, nestin, and vimentin, are all elevated following retinal injury, and that nestin and synemin may have functional significance in the remodeling process of reactive glial cells. Nestin, GFAP, and vimentin IF proteins are dramatically upregulated in Müller cells within a day of retinal damage, but the levels of each vary considerably among individual Müller cells, while synemin expression remains low until at least the 7 day time point. Previous studies have shown that GFAP expression increases greatly after retinal injury and our data suggest that nestin expression undergoes a similarly dramatic increase in Müller cells. Indeed, nestin is the predominant IF protein expressed in regions of BrdU labeled Müller cells, as well as in Müller cell processes extending outside of the neural retina. While synemin is also upregulated in Müller cells after injury, its increase in expression is the least dramatic of the four IF proteins studied here. Interestingly, in contrast to nestin, synemin is starkly upregulated by retinal astrocytes in response to injury; these cells develop long processes that extend from the NFL into the outer retina and that maintain a close association with blood vessels. Altogether, our observations indicate that in response to retinal injury, both nestin and synemin expression mirrors that of GFAP; all three are upregulated in Müller cells, and synemin is expressed by astrocytes as well. To our knowledge, this is the first demonstration that synemin is an indicator of reactivity to injury by retinal astrocytes. In fact, by comparison to Müller cell reactivity, astrocyte reactivity has not been well characterized in the retina, probably because structural changes in astrocytes are greatly overshadowed by the larger and more abundant changes found in Müller cells.

Tawk et al. [37] have shown the presence of synemin in Müller cell endfeet in normal and diseased human retinas, as well as in some neuronal processes in the inner nuclear layer. However, our observations strongly suggest that astrocyte labeling accounts for the synemin signal observed in the NFL. Nonetheless, our results and those of Tawk et al. [37] both demonstrate the presence of synemin in the Müller cells of injured and diseased retinas, in our case in an experimental model of retinal injury and in theirs in retinas from patients with Merkel’s syndrome.

While synemin is not present in normal mature astrocytes, it has been demonstrated in human reactive astrocytes in various diseases [38]. Histological preparations of cortical tissue have shown that anti-synemin labels reactive astrocytes in wild-type mice after entorhinal cortical lesioning, but not in GFAP and vimentin null mice [39]. Synemin was also absent in vimentin null mice, suggesting that vimentin is essential for synemin to accumulate at the protein level. GFAP and vimentin have long been known to be present in astrocytes throughout the course of development, but it is now realized that these cells also produce synemin and nestin en route to becoming mature glial cells [8]. In this context, our finding that following retinal injury Müller cells reexpressing both synemin and nestin also incorporate BrdU suggests that they may be reverting to an earlier developmental phenotype compared to the quiescent Müller cells found in normal retinas. Furthermore, the reexpression of nestin and synemin may be important for the proliferation of Müller cells in the injured retina, since nestin has been shown to facilitate the mitotic disassembly of the IF network [40], and has also been proved to be a positive regulator of astrocytoma cell proliferation [41]. In a companion study however, we could obtain no positive evidence for the dedifferentiation of mitotic Müller cells after injury using a variety of accepted progenitor markers [27]. Thus, the question of Müller cell dedifferentiation after retinal injury in mammals has not been conclusively answered.

Another discovery revealed by our immunocytochemical data is the striking variability among individual Müller cells with respect to their expression of various combinations of IF proteins. Although the functional significance of this variability is currently unknown, it was consistently observed from 1 day of injury. Müller cell reactivity following injury or disease has long been known [42–44], but the data presented here demonstrate additional features of the retina’s response to injury. First, Müller cells display cytoskeletal changes more diverse and complex than previously thought; second, our data demonstrate significant retinal astrocyte reactivity in response to trauma. These complex changes within Müller cells probably allow for the dramatic structural remodeling that occurs in these large, elaborate radial glia as they hypertrophy within the retina and form glial scars on retinal cell surfaces. Therefore, changes in the composition of the IF proteins may also be functionally associated with the reentry of many of these cells into the cell cycle, especially given the association between increased nestin expression and BrdU incorporation.

Previous studies have demonstrated that the absence of GFAP and vimentin greatly reduces the formation of glial scars elsewhere in the CNS, as well as in the retina [30,45]. As elsewhere in the CNS, rapid changes in retinal glial cells may have both beneficial and detrimental effects on the return of function, a subject that remains controversial. Certainly, glial scar tissue formed by Müller cells can thwart photoreceptor outer segment regeneration [23] or become contractile, causing wrinkling or even reinjuring the retina by subsequently causing its separation from the RPE. However, the overall effect of glial reactivity on retinal healing after injury is in need of additional studies.
